# Biodiversity in remnants of natural mountain forests under conservation-oriented management

**DOI:** 10.1038/s41598-018-35448-7

**Published:** 2019-01-14

**Authors:** Jakub Horák, Jan Materna, Josef P. Halda, Strahinja Mladenović, Petr Bogusch, Pavel Pech

**Affiliations:** 1 Faculty of Forestry and Wood Sciences, Czech University of Life Sciences Prague, Kamýcká, 1176, CZ-165 00 Prague, Czech Republic; 2 Krkonoše National Park, Dobrovského 3, CZ-543 01 Vrchlabí, Czech Republic; 3 University of Hradec Králové, Faculty of Science, Department of Biology, Rokitanského 62, CZ-500 03 Hradec Králové, Czech Republic

## Abstract

The structure of forests is an important stabilizing factor regarding ongoing global climate and land use change. Biodiverse mountain forests with natural structure are one of the ecosystems most endangered by these problems. We focused on the mountain forest islands of European beech (*Fagus sylvatica*) and their role in the natural distribution of organisms. The study area was situated in the oldest Czech national park, Krkonoše (385 km^2^), which is the highest mountain ridge in the country. We studied multi-taxa (lichens, beetles and hymenopterans) responses to three hierarchical spatial levels of the environment: the topography was described by the elevation gradient; the patch structure was described by canopy openness, dead wood amounts, and Norway spruce (*Picea abies*) cover; and the tree level was described by species of the sampled tree and its diameter. Lichens preferred higher elevations, while insect groups responded conversely. Furthermore, insect groups were mainly influenced by the inner patch structure of beech islands. Lichens may be jeopardized due to the predicted future increase in temperatures, since they would need to shift toward higher altitudes. Insects may be mainly threatened in the future by land use changes (i.e., forest management) – as indicated by an interconnection of canopy openness and the amount of dead wood.

## Introduction

Forest structure is an important factor regarding future global changes^1^. Specifically, due to the ability of forests to influence the local climate and their importance as a global carbon stock and in providing biodiversity^2^. Even though some forests benefit from sustainable development policies, many governments are trying to improve their environment using subsidies^3–5^.

The situation of forests in Europe appears to be relatively promising with respect to their biodiversity in the future. However, their historical abuse makes their preservation difficult^6^ – at least one tenth of the forests of Central Europe consist of plantations dominated by Norway spruce (*Picea abies*)^7^. The main reason for the dominance of spruce plantations in Europe and their preference by foresters is because of their economic value. Forests dominated by this conifer are usually considered to have lower biodiversity than the deciduous forests that once dominated the mainland of Central Europe^8,9^. Thus, one of the most important global actions is the future change of conifer monocultures into stands that support native trees or at least into mixed stands^10,11^. Nevertheless, European beech (*Fagus sylvatica*) forests are still relatively common in the higher elevations of Central Europe^12^.

Approximately 10% of the forest area in Europe is in conservation areas^13,14^. Nevertheless, an increase in area of conservation appears not to be the only remedy to protect forest biodiversity. While some authors concluded that conserved forests have higher biological diversity than managed plantations^15,16^, others have found that some taxa or species profit from human-managed forests^9,17,18^.

Insects and lichens are some of the most-used taxa for the evaluation of forest ecosystem conditions. It is known that most lichens are dependent on veteran trees^19^, which are an important element of biodiverse forests^20^. The species richness of lichens is also known to be higher when the forest landscape is more heterogeneous, and a similar situation occurs with insects^21^. In European forest conditions, a long period of time is necessary for lichen species to inhabit a suitable area – up to 200 years is suggested to be enough for the creation of a sustainable population of lichens^22,23^. The amount and diversity of dead wood are other important parameters for lichens, as they are known to prefer dead wood in early stages of decay^24,25^. To support threatened lichen species in mountain areas, it is also recommended to enrich spruce stands with broadleaf tree species^26^.

Beetles and hymenopterans are among the most species-rich forest taxa. It is often mentioned that managed forests sustain fewer insect species compared to virgin forests^27,28^. One of the most common factors that negatively affects arthropods in forest ecosystems is the total habitat transformation from native tree species to commercial species that are cost effective^29^. It is crucial to obtain more data regarding taxa that are sensitive to different management systems or to abandonment at all^30^. Some of the most important management-sensitive taxa in forest ecosystems are lichens, as they are sedentary organisms with a possible preference for close-to-natural forests, and insects, as they are more mobile taxa with a preference for disturbance.

Our interest was to investigate the role of natural mountain beech (*Fagus sylvatica*) forest islands in the distribution of organisms. We selected three hierarchical levels of the environment that potentially influenced three selected taxa. Specifically, we studied the response of lichens, beetles, and hymenopterans to the topography, forest island patchiness, and individual tree characteristics in the highest mountain ridge in the Czech Republic, Krkonoše. This mountain ridge is part of the oldest Czech national park and is the most isolated within Europe. We also searched for environmental thresholds that may be important for isolated forest islands.

## Methods

### Study area

Krkonoše is a 631 km^2^ area in the Czech Republic and Poland. It is the oldest national park (est. 1963) and the highest mountain area (Sněžka with 1,603 m a.s.l.) in the Czech Republic, with an area of 385 km^2^. Quaternary glaciers influenced its morphology and created extensive plateaus without forest cover specific to the highest mountains in Europe. These plateaus in Krkonoše are partly composed of Dwarf pine (*Pinus mugo*) vegetation at the upper limit of woody vegetation. At the upper tree limit, the vegetation of Dwarf pine is replaced by Norway spruce. The upper limit for forests in Krkonoše is 1,200–1,350 m a.s.l. Forests here are dominated by Norway spruce but also include European beech or mixed forests^31^.

This mountain ridge started to be affected by humans at least by the 7^th^ century. From the 14^th^ century, this ridge was mainly affected by extensive deforestation (mainly beech forests) to supply timber for industry in lowlands. The 18^th^ century brought deforestation due to new permanent settlements at high elevations. Forests were then affected by industrial air pollution starting in the middle of the 20^th^ century. This gave rise to the present state of the dominance of Norway spruce stands (79%) partly mixed with the studied natural islands of beech-dominated vegetation^12,31^.

### Sampling of taxa and environment

**Figure 1:**
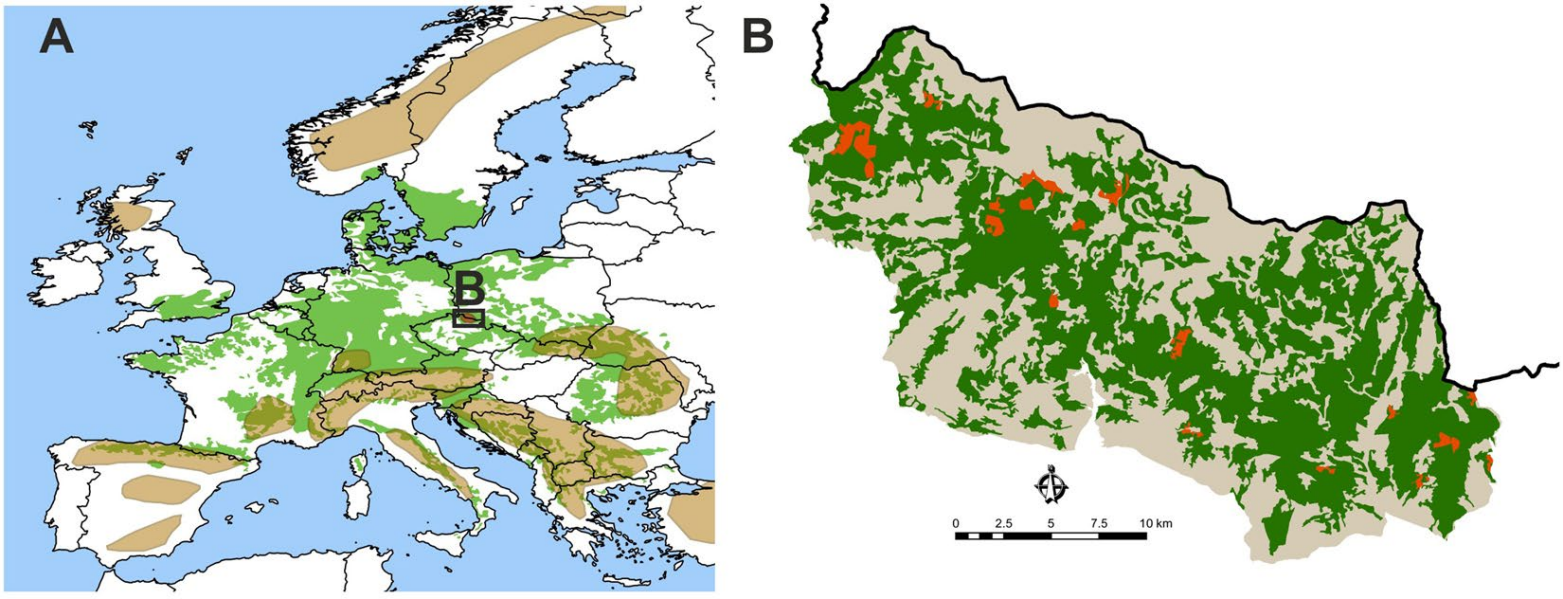
Visualization of the studied area. (**A**) Distribution of mountain beech forests (green) and overlap with highest mountains (brown) in Europe. The study area of Krkonoše (gray) is highlighted by the black frame. (**B**) Distribution of forest cover in Krkonoše is green, studied beech islands are orange.

We sampled 128 forest patches (10 m radius surrounding a target tree) inside 16 beech islands (mean = 56.4 ± 5.7 SE ha) distributed in Krkonoše (Fig. 1) using equal-stratified sampling^32^ by studying 8 patches per beech island. All beech islands were isolated from each other by forests dominated by Norway spruce. We used a multi-taxa approach; we studied three taxa with different environmental demands. Lichens were visually sampled at 128 target trees at the end of July 2014. Insect taxa of beetles (Coleoptera) and aculeate hymenopterans (Hymenoptera: Aculeata) were sampled using cross-panel trunk tree traps fixed on the south side of each target tree^33^. They were sampled from May to September in 2013 for the 64 patches in the east-central part of Krkonoše and in 2014 for the 64 patches in the west-central part.

We were interested in the influence of the environment at three spatial levels: (i) topographic, (ii) patch, and (iii) tree. Altitude (800.8 ± 12.32 SE; 571.7–1079.4 m a.s.l.) was studied as an important topographical variable reflecting the elevation gradient, which is important for understanding possible shifts in the distributions of species due to climate change^34^. Altitude was not affected by different aspects and the possible effect of gradient inversions, because forests in the Czech part of Krkonoše consist mainly of south-facing slopes.

Three patch level variables were also studied at a 10-m radius surrounding a target tree^35^. The amount of dead wood (2.2 ± 0.3 SE; 0.1–20.0 m^3^) was measured as a reflection of natural disturbances and the sustainable development of forests. Openness in the canopy (12.6 ± 0.5; 4.1–33.8 SE %) was measured as the reflection of gradient of overstory disturbance that influences the microclimate independent of altitude^36^. The canopy was photographed with a Cannon EOS 600D with Sigma circular fish eye DC HSM 4.5 mm 1:2.8 (180° angle) lens at four places in the patch at 1.5 m above the ground. The first two pictures were taken at the top of the trap. The next three pairs of pictures were taken at 5 m from a target tree: the first pair at the north side and the other two pairs at the corners of an equilateral triangle. Pictures were evaluated in GLA 2.0. The cover of spruce (40.3 ± 2.4 SE; 0–100%) in the tree species composition of the overstory was measured as the percentage of this tree species in the crown area of the patch. This was the reflection of artificial plantings and natural regeneration by this conifer tree within beech islands.

We also studied two tree-level characteristics^37^ – i.e., the effect of the subject. Specifically, we measured the diameter at breast height (DBH; 49.3 ± 1.1 SE; 28.5–84.6 cm) as a reflection of the potential habitat area for species attracted by the target tree^38,39^. We studied beech (N = 64) and spruce (N = 64) in pairs (i.e., 4 pairs of spruce and beech per forest island). Only mature trees were used as target trees, and we used trees without microhabitats (e.g., tree hollows, conks of fungi or dead limbs).

### Statistical analyses

All analyses were done in R 3.0.2.

Dependent variables (species richness of the studied taxa) were first tested for potential spatial bias (using the spdep package) using Moran’s I, which was not found for all studied taxa: lichens (I = −0.01; P = 0.71), beetles (I = −0.01; P = 0.08) and aculeate hymenopterans (I = −0.01; P = 0.36).

Independent predictors were tested for potential multicollinearity (package HH) using a criterion of VIF < 2, and multicollinearity was not found. The studied beech trees were used for statistical analyses on a semi-quantitative scale and coded as 1, while spruce was coded as 0.

Hierarchical partitioning (package hier.part), a method that informs the explained variance of particular independent variable, was used to compute the independent (by a particular independent variable) and shared explained variance (variance shared with other independent variables).

Initial generalized linear models (GLMs) that included all independent variables were computed with the appropriate distribution (a Poisson distribution for lichen species richness, a Gaussian distribution for square root-transformed beetle species richness, and a quasi-Poisson distribution for aculeate hymenopterans).

Next, GLMs were selected (packages MASS, pgirmess, and nlme) from those that met the criterion of ΔAICc < 2. A χ^2^ test was used for the comparison of these models with the best subset model (i.e., the model with the lowest AICc). The differences in AICc did not drop significantly, and thus, the best subset model for each taxon was used for partial regressions.

Partial regressions (package car) of the best subset GLMs were then computed and visualized using Pearson residuals. Visualization illustrates the relationship between dependent and independent variables, with interaction with all other independent variables used in the best subset GLMs.

The conditional inference tree method (package party), from the family of recursive partitioning based on maximally selected rank statistics, was used for the selection of threshold values of independent variables.

Bootstrapping (N = 1000; package boot), a method that allows measures of accuracy to be assigned (defined in terms of confidence intervals) to sample estimates, was used for the computation of 95% confidence intervals of thresholds.

## Results

We observed 37 species of lichens, and trapped 286 species of beetles and 37 species of aculeate hymenopterans.

### Variance explained by independent variables

**Figure 2:**
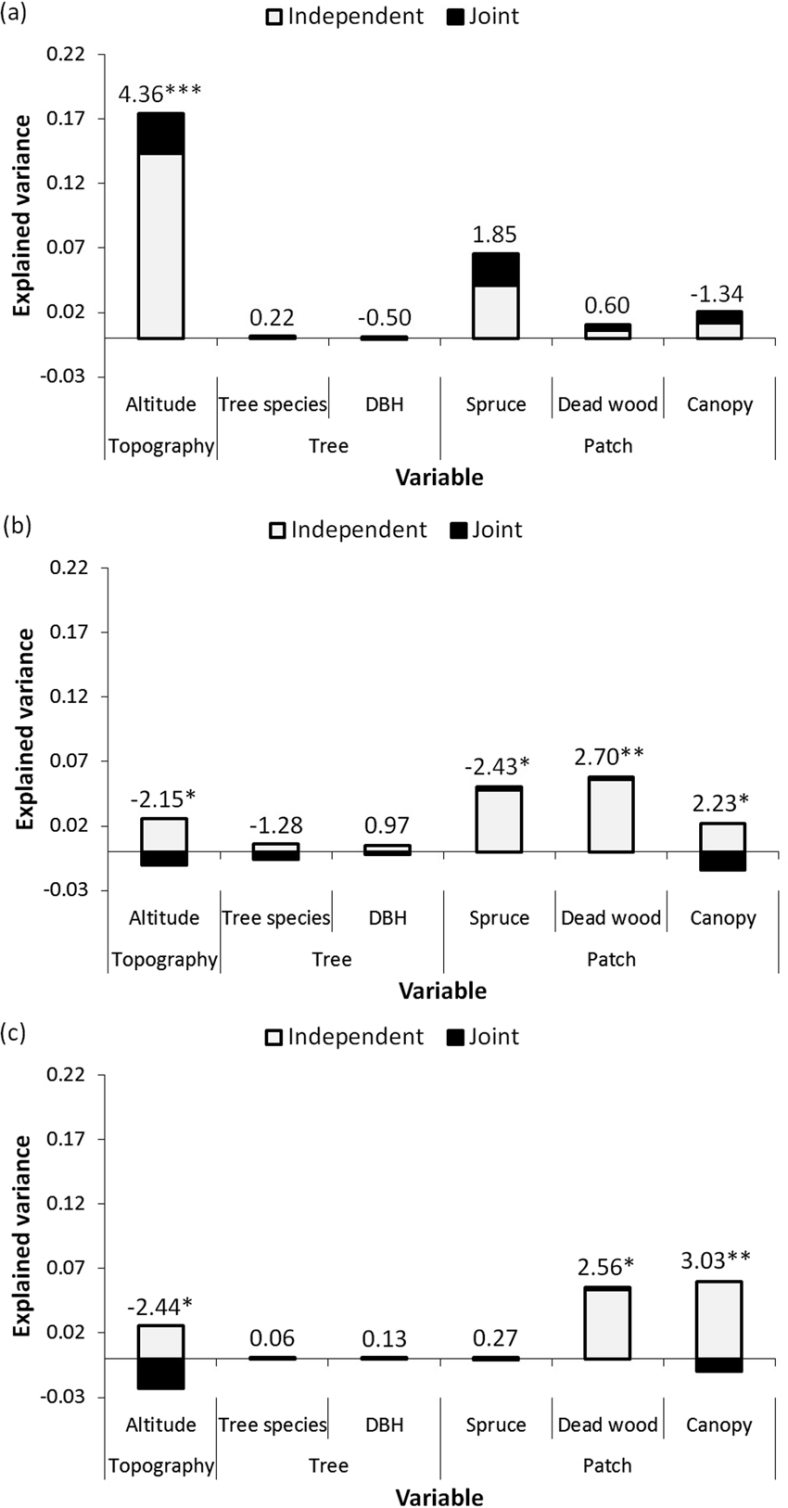
Multi-taxa species richness responses to the environment in Krkonoše National Park in the Czech Republic. The results of hierarchical partitioning (Y-axes) and generalized linear models are visualized for (**a**) Lichenes, (**b**) Coleoptera, and (**c**) Aculeata Hymenoptera; t- or z-values above bars together with significant P-values of GLM are indicated by *For P < 0.05; **For P < 0.01, and ***For P < 0.001. Note that a predictor with a negative shared contribution indicates that other predictors act as its suppressors.

Altitude explained the highest proportion of variance in the case of lichens. This variable was followed by the representation of spruce, but its variance shared with other independent variables was relatively high (Fig. 2a). Spruce and dead wood, followed by altitude and canopy openness, were the independent variables that explained the highest variance in the case of beetles (Fig. 2b). Dead wood and canopy openness were the most influential independent variables regarding the explained variance in the case of aculeate hymenopterans. These two variables were followed by altitude (Fig. 2c). The total and independent effects of other independent variables on species richness were rather low. Canopy openness and altitude were the most negatively influenced by interaction with other independent variables in the case of insect taxa (Fig. 2).

### Initial GLMs

Lichens were significantly influenced by topography and increased in species richness toward higher elevations (Fig. 2a). Beetles had the most complex response pattern and were significantly influenced by topography and all studied patch variables. Specifically, their species richness was promoted by increasing the amount of dead wood and openness in the canopy, whereas the effect of an increasing amount of spruce and increasing altitude negatively affected this taxon (Fig. 2b). Aculeate hymenopterans also had a complex response to the environment. They were significantly influenced by topography and some of the patch characteristics, namely, the number of species was negatively affected by rising elevation, while the amount of dead wood and canopy openness had positive effects (Fig. 2c). We did not observe any effect of the subject –, i.e., the target tree on the studied taxa (Fig. 2).

### The results of GLM selection

The results of model selection revealed that altitude was the most influential characteristic, included 11 times in the final 12 models selected for all studied taxa. Regarding the patch parameters, spruce cover was present eight times, and canopy openness and dead wood were present seven times. Tree-level characteristics were included only three times for DBH and twice for the target tree species (Table 1).

**Table 1:** Characteristics of the GLM selection based on data from Krkonoše National Park in the Czech Republic. Best subset GLMs and others with Δ AICc <2 are listed and ordered by their AICc.

Taxa	Variables	AICc	ΔAICc	P
Lichenes	Altitude + Spruce	565.24	0	—
	Altitude + Spruce + Canopy	565.80	0.56	n.s.
	Altitude	565.94	0.70	n.s.
	Altitude + Canopy	566.87	1.63	n.s.
	Altitude + DBH + Spruce	567.16	1.92	n.s.
	Altitude + Spruce + Deadwood	567.17	1.93	n.s.
Coleoptera	Altitude + Spruce + Deadwood + Canopy	304.42	0	—
	Altitude + Tree + Spruce + Deadwood + Canopy	304.71	0.29	n.s.
	Altitude + DBH + Spruce + Deadwood + Canopy	305.43	1.02	n.s.
	Altitude + Tree + DBH + Spruce + Deadwood + Canopy	305.99	1.57	n.s.
	Spruce + Deadwood	306.29	1.88	n.s.
Aculeata	Altitude + Deadwood + Canopy	453.72	0	—

P-values are for possible significant drops of ΔAICc.

**Figure 3:**
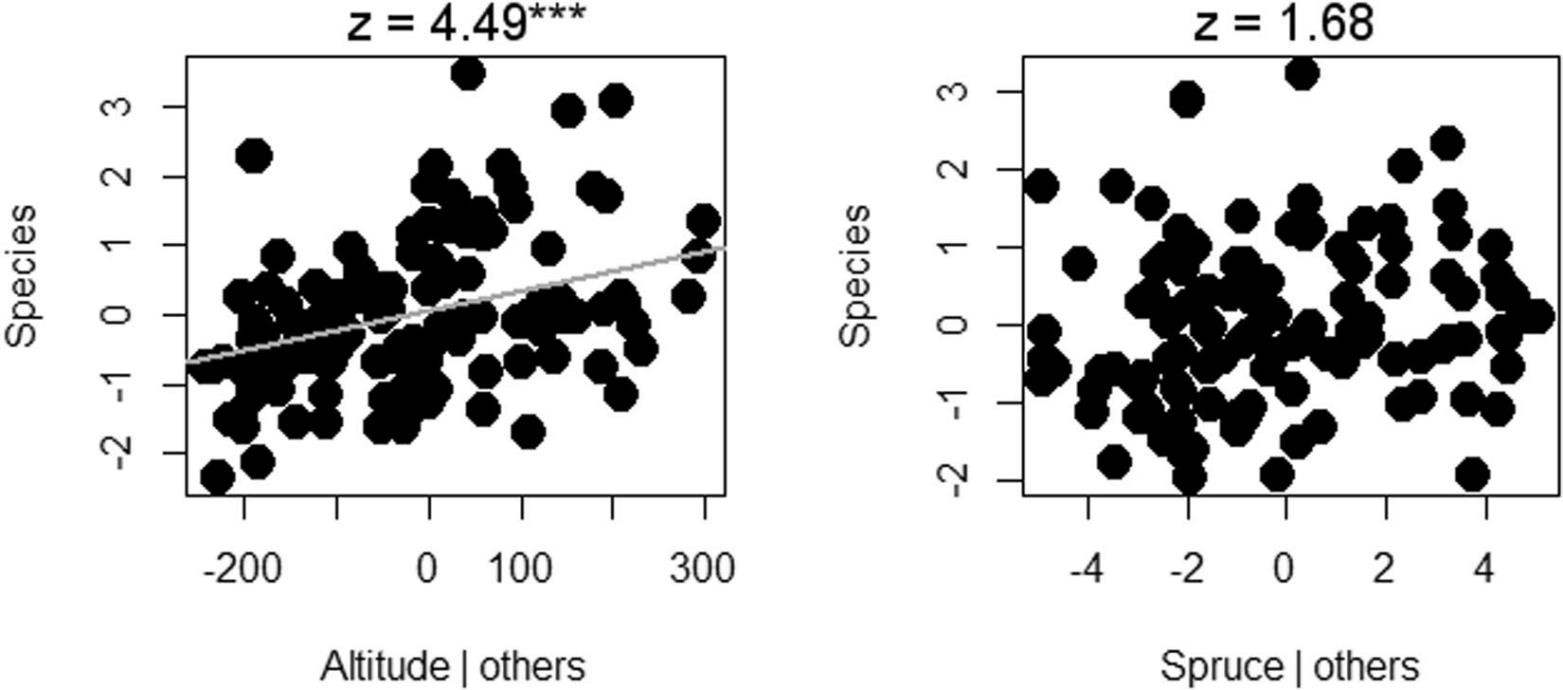
Lichenes species richness response to the environment in Krkonoše National Park in the Czech Republic. The results of partial regression of the best subset of the GLM are visualized by Pearson residuals and gray regression lines for significant responses; z-values together with significant P-values of the GLM are indicated by ***For P < 0.001.

**Figure 4:**
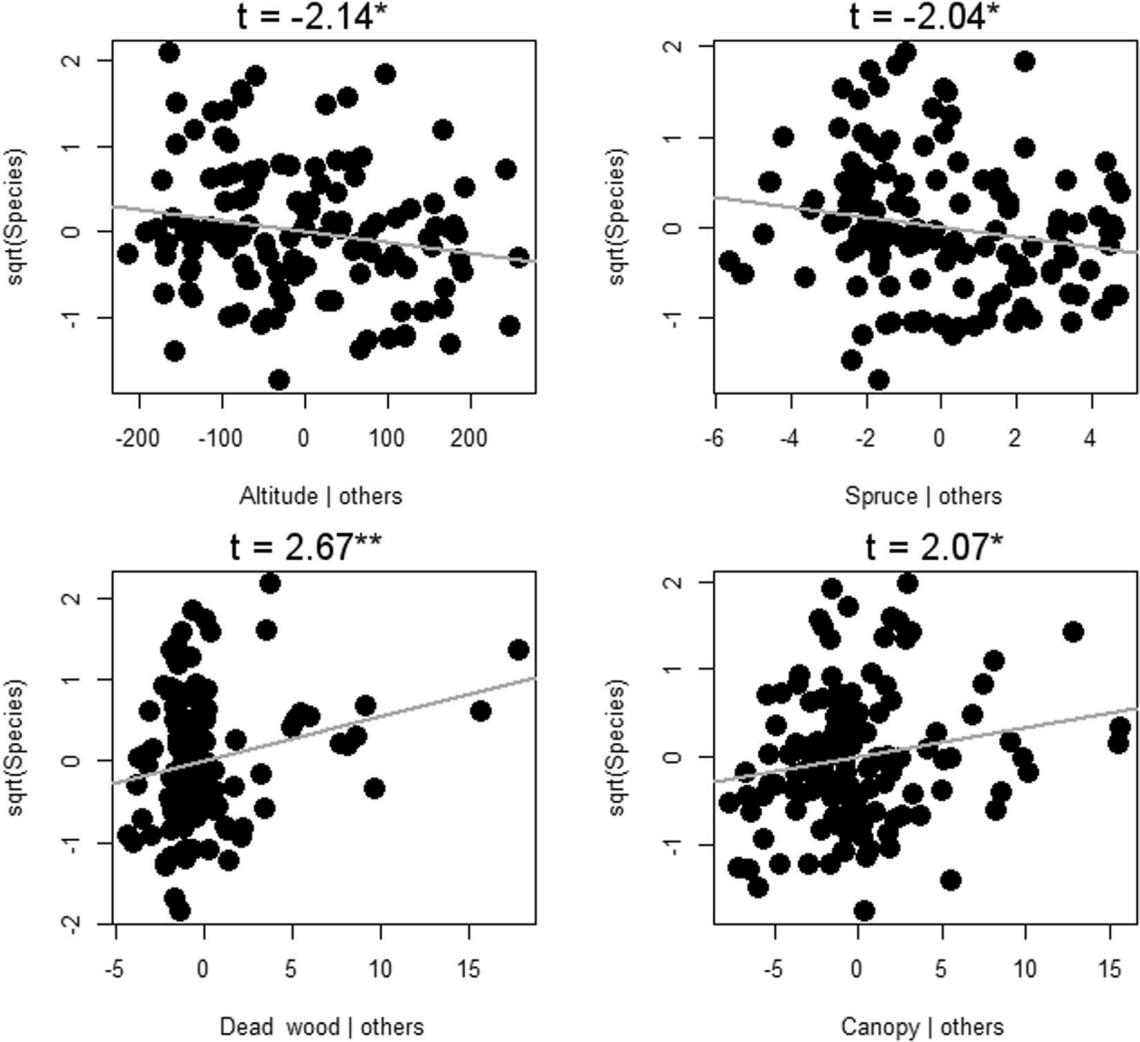
Coleoptera species richness response to the environment in Krkonoše National Park in the Czech Republic. The results of partial regression of the best subset of the GLM are visualized by Pearson residuals and gray regression lines for significant responses; t-values together with significant P-values of the GLM are indicated by *For P < 0.05 and **For P < 0.01.

**Figure 5:**
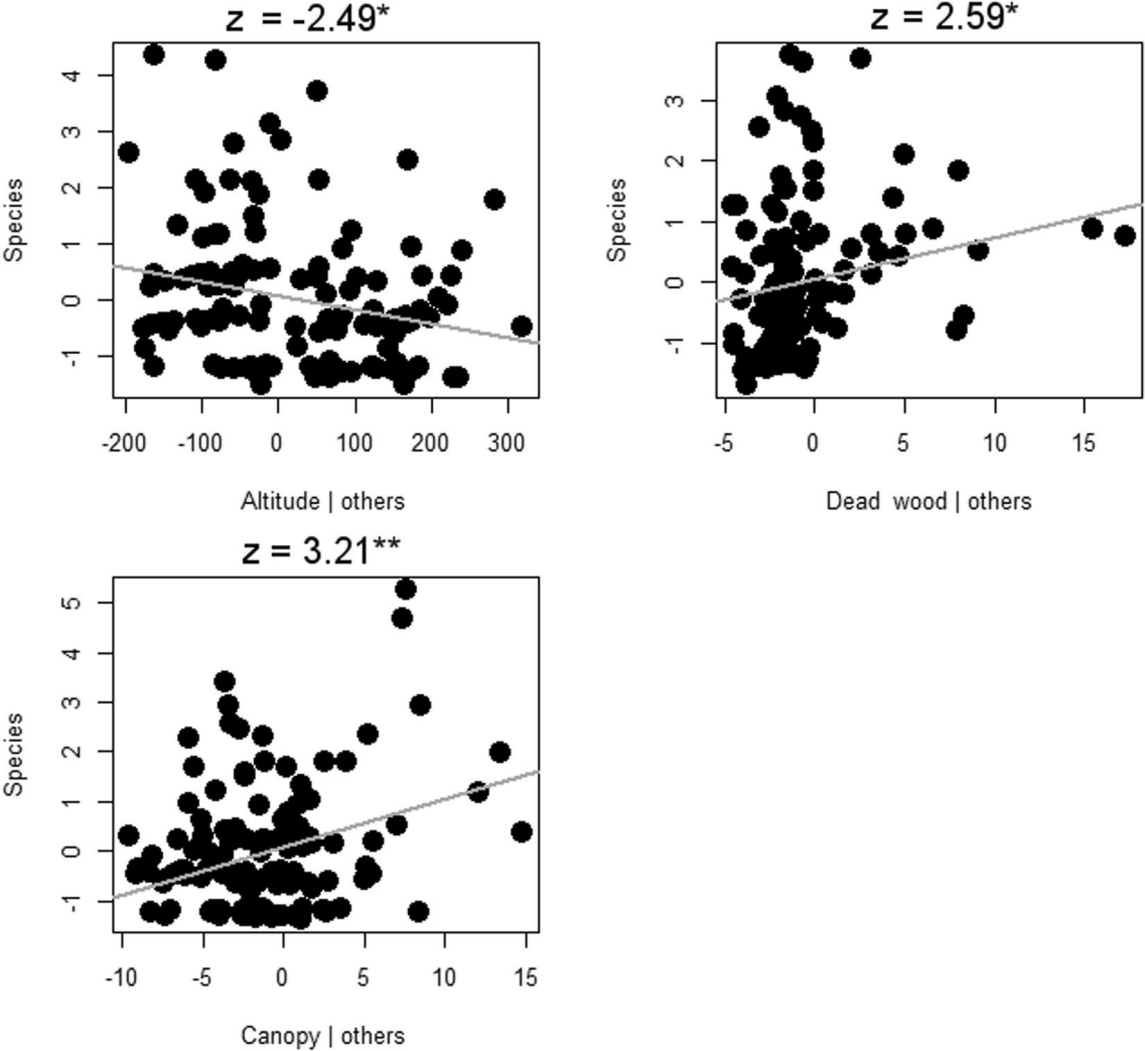
Aculeata Hymenoptera species richness response to the environment in Krkonoše National Park in the Czech Republic. The results of partial regression of the best subset of the GLM are visualized by Pearson residuals and gray regression lines for significant responses; z-values together with significant P-values of the GLM are indicated by *For P < 0.05 and **For P < 0.01.

The best subset model indicated a significant influence of increasing altitude on the number of species of lichens. The cover of spruce also stayed in the best subset model, but it had no significant effect (Fig. 3). Beetles were negatively affected by increasing elevation and spruce cover, while rising dead wood amounts and openness in the canopy had positive effects (Fig. 4). Aculeate hymenopterans had the same response as beetles, except there was influence from spruce only on beetles (Fig. 5).

### Thresholds

**Figure 6:**
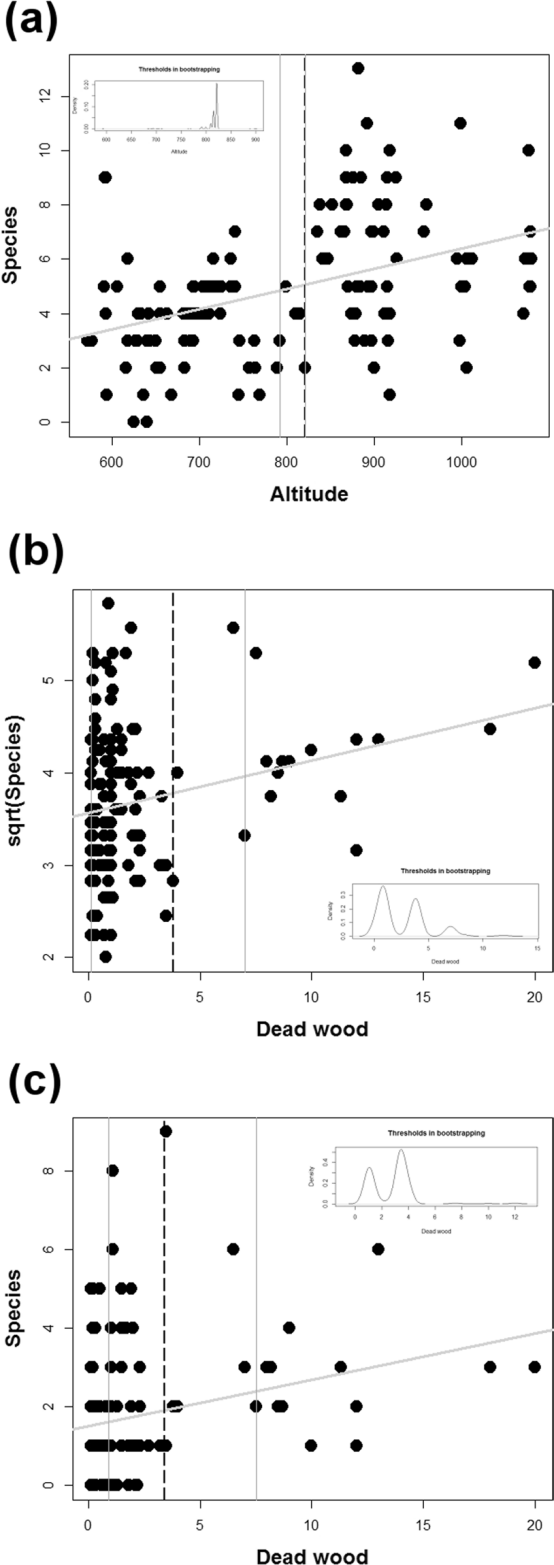
Multi-taxa species richness response to the most important predictor in Krkonoše National Park in the Czech Republic. The results of conditional inference tree methods and bootstrapping are visualized for (**a**) Lichenes, (**b**) Coleoptera, and (**c**) Aculeata; black dashed vertical lines are significant threshold values; thin gray vertical lines are 95% confidence intervals; thick gray lines are linear regressions; and inset frames are the density plots of all thresholds found using bootstrapping.

Altitude was the only factor that indicated a threshold value for lichen species richness. The threshold was 821.1 m a.s.l., above which the number of species was significantly higher than below. The mean number of species above this altitude was 6.28 at 60 sites, while the mean below this altitude was 3.74 species at 68 sites. The majority of bootstrapped thresholds (95% confidence interval) were between 800 and 825 m a.s.l. (Fig. 6a). The dead wood gradient indicated thresholds for insect taxa. Beetles had a threshold value of 3.8 m^3^, and the mean species value above this threshold was 17.74 in 17 sites, while the mean under this value was 13.74 at 111 sites. Aculeate hymenopterans had a lower threshold, at 3.4 m^3^ of deadwood, with a mean species value of 3.05 in 19 sites above and a mean of 1.55 below this threshold value at 109 sites. Density plots of bootstrapped thresholds and 95% confidence intervals are visualized in Fig. 6b for beetles and in Fig. 6c for aculeate hymenopterans.

## Discussion

We studied the response of biodiversity to the environment in natural beech islands using a multi-taxa approach. Insect taxa were negatively affected by increasing elevation, while lichens showed the opposite response. Given that lichens are highly sedentary and sensitive to changes in the environment, our results suggest that they can become threatened by the future climate change scenario. However, high elevation areas in mountains without forest cover still represent some possible chance for survival of these lichen forest associates. It is questionable whether the shift of their habitat (i.e., beech forest) toward higher elevations would respond to climate change as quickly as needed for the migration of the lichens. The problem is that some species (e.g., *Graphis scripta* and *Trapelia corticola*) depend on the presence of old trees and sometimes also ancient woodland structures^40^. Thus, their migration lags at least hundreds of years behind the establishment of forest vegetation^41^. Because the dispersal rates of lichens are highly limited, the only chance for their survival in the future appears to be shifts in the ecological requirements of individual species, which is not common but has been observed even in species close to extinction^42^. Nevertheless, because the lichens were significantly influenced only by elevation, we can conclude that this highly isolated mountain ridge might be a good natural laboratory for the study of the possible effects of climate change in the future. Moreover, the threshold value for altitude corresponds with the mean altitude in this study and is also an approximate border between the sub-montane and montane forests in Krkonoše and elsewhere in Central Europe.

Insects were negatively affected by the increasing altitudinal gradient and may not be directly threatened by the ongoing climatic change scenario. Insect taxa were mainly influenced by the patchy structure of beech islands. Thus, they are potentially mainly affected by the increase in changes in patch structure of isolated beech islands. The main potential threat for insects probably comes from a decrease in dead wood connectivity. The observed threshold values (converted per hectare) for dead wood amounts (108.3–121.0 m^3^/ha) were relatively optimistic from the point of view of similar forest habitats in Europe^43^, yet they were still lower than in the oldest Czech forest reserves^35^. The need for higher dead wood amounts for saproxylic insects in higher elevations is well known^44^. In our case, dead wood amounts reflected natural disturbances and the sustainable development of forests rather than habitat availability, as we did not study only the saproxylic guild. However, it was surprising to observe a positive response to higher canopy openness combined with higher dead wood amounts^44^ for both studied insect taxa. The interconnection of the positive effect of increasing dead wood amounts together with the increase of canopy openness (i.e., disturbance causing an increase of local temperatures) is more surprising, since these two environmental variables were not collinear.

The contamination of beech islands by spruce is very important information from the European perspective. This question was important for our research due to the possible influence of spruce on biota in medium and lower altitudes, where this tree is not indigenous. This species is indigenous to the highest parts of mountain forests in Krkonoše^31^. However, its artificial planting in the past makes this species the most important and widespread forest tree in the majority of the country, and the same situation is found in Central Europe. Moreover, Norway spruce is one of the most endangered tree species regarding local increases in temperature^45^. Hence, one of the possible problems is its ongoing planting in lower altitudes^46^. Thus, the management activities intended to decrease the amount of spruce (i.e., artificial interventions in the unmanaged beech forest islands) are important due to the affectation of beetles to increasing spruce cover.

This appears to be an important issue not only from the perspective of mountain ridges but also for other similar tree species through the world. First, Norway spruce is a possible biological contaminant elsewhere in Central Europe; second, spruce might be negativelly affected by the possible increase of temperatures and decrease in precipitation; and third, this could be an example of how important the influence of non-indigenous species on native biota can be. From the first perspective, we can conclude that the spread of spruce might be a threat to beetles. Based on the second perspective, spruce admixture and the creation of mixed mountain forests in the future is probably the only option to preserve the native spruce-associated biota, since stands with a dominance of spruce may disappear in the future climate scenario^47,48^. From the point of view of the third perspective, the removal of the non-indigenous trees in areas of high conservation interest may help biodiversity and conservation.

We did not observe any effect of the subject (i.e., influence of the characteristics of target tree), which is a relatively surprising issue regarding future methodological possibilities. We used only those trees that do not bear any visible microhabitats as target trees^20,37^, which probably led to this result. This also showed us the suitability of tree trunk traps instead of those used outside the tree. Specifically, it can be seen that the recorded tree factors did not bias the results. This is also important, as it is known that tree trunk traps are suitable for communities of insects that are less mobile and more specialized, namely, often-trapped flightless species^38,39^, which have a strong ability to indicate forest fragmentation^49^. No effect from the subject is also important for future studies that may show us possible temporal change due to climate change, especially because beech trees in mountainous areas will probably grow faster (i.e., increase their DBH) in the future, allowing the use of the same sites for comparison. Nevertheless, it seems that the DBH would play a role even when the tree dies, thereby increasing the amount of dead wood (i.e., during the afterlife of a tree)^50^.

### Implications for management

From the point of view of the two insect taxa studied, it seems that future climate change, together with low-intervention forest management, would have a positive impact on their species richness. This result needs to be taken with caution, as the response of lichens is not so optimistic.

We can conclude that the most suitable management would be the selective cutting of spruce trees. In addition to lowering the amount of spruce, this option would increase the canopy openness and dead wood amount. This management option should be implemented with the leaving of beech dead wood inside the islands. The natural decline of spruce in the future is also one of the possible options, effectively a hands-off management of beech islands. A majority of spruce trees would die, the canopy would be more open, and the amounts dead wood would increase. Thus, the interaction of hands-off management and climate change might have a positive effect on insects. However, this is a solution only from the point of view of the insect taxa. The solution for lichens is not so simple, and probably, the only management should be based on the resignation of cutting of carefully selected trees, allowing them to reach the proper conditions of veteran trees.
